# The fundamentals of eye tracking part 4: Tools for conducting an eye tracking study

**DOI:** 10.3758/s13428-024-02529-7

**Published:** 2025-01-06

**Authors:** Diederick C. Niehorster, Marcus Nyström, Roy S. Hessels, Richard Andersson, Jeroen S. Benjamins, Dan Witzner Hansen, Ignace T. C. Hooge

**Affiliations:** 1 Lund University Humanities Lab and Department of Psychology, Lund University, Lund, Sweden; 2 Lund University Humanities Lab, Lund University, Lund, Sweden; 3 Experimental Psychology, Helmholtz Institute, Utrecht University, Utrecht, the Netherlands; 4 Tobii AB, Danderyd, Sweden; 5 Experimental Psychology, Helmholtz Institute & Social, Health and Organizational Psychology, Utrecht University, Utrecht, the Netherlands; 6 Eye Information Laboratory, IT University of Copenhagen, Copenhagen, Denmark

**Keywords:** Eye tracking, Tools, Software, Programming, Hardware and

## Abstract

Researchers using eye tracking are heavily dependent on software and hardware tools to perform their studies, from recording eye tracking data and visualizing it, to processing and analyzing it. This article provides an overview of available tools for research using eye trackers and discusses considerations to make when choosing which tools to adopt for one’s study.

## Introduction

This article is the fourth in a series on the fundamentals of eye tracking (see also Hessels et al., 2025; Hooge et al., in press; Nyström et al., in press). The articles are aimed at individuals who are (one of) the first in their group, company, or research field to use eye tracking, with a focus on all the decisions one may make in the context of an eye-tracking study. Such individuals may come from academia (e.g., psychology, biology, medicine, educational science, computer science), commercial institutions (e.g., marketing research, usability, decision making) and non-commercial institutions (e.g., hospitals, air traffic control, military organizations). Note that this is not an exhaustive description of the target audience. More experienced eye-tracking researchers may find useful insights in the article series, or may find the article series a useful reference or hub to relevant research. One may either choose to read this article as the fourth part of the series, but one may choose to skip the other articles in the series if this article is of more immediate interest.

This article discusses what researchers use to collect, visualize, process, and analyze eye tracking data. We will refer to the software (and hardware) researchers use for these tasks as eye tracking tools. In this article, we will provide an overview of different tools that can be used for eye tracking research, and provide advice on choosing tools that fit one’s operation. A notable exception in this discussion is the eye tracking hardware itself, which is instead discussed in Nyström et al. (in press). For discussions of how eye movements and eye tracking can be applied in research, the reader is referred to Duchowski (2007); Holmqvist et al. (2011); Leigh and Zee (2015) as well as parts 1 and 2 in this article series (Hessels et al., 2025; Hooge et al., in press).

How are tools used by researchers when conducting eye tracking research? Imagine two extreme approaches that may be taken by researchers. At one extreme we find a person relying exclusively on commercial hardware and software, who we refer to as the proprietary software researcher. Often, eye tracker manufacturers also sell a software suite to go together with their eye trackers (e.g., SR Research Experiment Builder and Data Viewer, SMI Experiment Center,^1^ Gazepoint Analysis and Tobii Pro Lab) and there are also software suites available from companies that do not make their own eye trackers (e.g., Blickshift, iMotions). Such software suites are designed to take care of many of the difficult problems in doing research with eye trackers, from building experiments and presenting stimuli to visualizing the recorded data and performing all manner of analyses on eye tracking data. An advantage of software suites is that they can be used to carry out most mainstream research (such as reading research, various paradigms from experimental psychology, or usability research). Another advantage is that such software is essentially plug and play: many difficult problems (e.g., data synchronization, trial segmentation and fixation classification) that researchers may not even be aware of, or are interested in, are solved for them and almost everything necessary to conduct a study can be performed in the software. Despite being excellent tools for many researchers, proprietary software also has disadvantages, such as that its methods are not open-source and thus cannot be inspected for correctness. Furthermore, being heavily reliant on a single software suite may have several downsides. For instance, it is possible that an old experiment cannot be opened, run, or analyzed anymore when the developer performs a major update or drops support for a needed feature. Also, when the software does not fits one’s needs, one may have to look for solutions outside the software or invest time in learning possibly complicated extension procedures. If you prefer that your tools handle all the technical details and other difficult problems for you, then such all-in-one software suites are probably the simplest and most convenient way to conduct good research.

On the other end of the spectrum there is a person with technical skills and enough time, motivation and reasons to develop their own tools, who we refer to as the do-it-yourself (DIY) researcher. The authors of this article belong to this category and make a lot of their tools themselves (e.g., Agustin et al., 2010; Nyström & Holmqvist, 2010; Hooge and Camps, 2013; Hessels et al., 2017; Benjamins et al., 2018; Hansen et al., 2019; Niehorster et al., 2020a; Niehorster & Nyström, 2020; Niehorster et al., 2023). This type of researcher often conducts research that is not fully served by existing tools, and has to do some significant development themselves. They would, for instance, have to build 1) a stimulus-creation program; 2) a program for presenting the stimuli that can record synchronized gaze data at the same time; and 3) a fixation classifier for determining the on- and offsets of fixations. They may even need to develop custom hardware, and many other things. An advantage of the DIY approach to research is that there are few limits to the experimental setups and designs one can build and the analyses that can be performed: when tools are needed that do not exist, one creates them. Other advantages of inhabiting this side of the spectrum include having full control over and intimate knowledge of the experimental setup and analysis procedures, and that the setup may become simpler because all tools are exactly tailored to the research project without being burdened with extra unneeded functionality or with workarounds that are needed because existing tools do not exactly fit the needs of the research. There are, however, also disadvantages to the DIY approach. Firstly, it is skill-intensive because it requires good programming competence, an understanding of hardware and possibly electronics, good knowledge and skill in physics and mathematics, etc. The approach is also time-consuming, especially because tool development may become an open-ended problem with no guarantee of success. A final important potential disadvantage is that developing tools and spending a significant time programming should match one’s interests. For a researcher who already spends all their time and intellectual capacity on a difficult scientific problem, maybe spending additional time on building the tools is not feasible or worth it. Perhaps a leading motivation for becoming a DIY researcher is to be able to perform science beyond existing limits, and such researchers happily accept the extra effort such an approach demands.

Most researchers probably find themselves somewhere on the spectrum between the two extremes of the proprietary software researcher who does most of their research using a single software suite and the DIY researcher who builds everything themselves. For instance, there probably are a lot of researchers who use the simplest tools for the job and mix both approaches when designing their workflow for a study. They happily use their favorite software suite where possible, but may choose to use another tool or even develop their own tool when they encounter a problem that cannot be solved using their preferred software suite. One can also choose to handle generic or difficult parts of the research workflow, such as stimulus presentation and fixation classification, using one’s standard tool, but choose to develop oneself the parts of the analysis that are heavily tied to the research question. Such researchers reap the benefits of being able to quickly execute a lot of their work using tools they know well, while their skills and knowledge of other available tools enable them to not be limited by their standard tools when the research problem demands more.

There are many good open-source and paid tools around that can do the difficult jobs for the researcher. However, how does one find and select the tools needed to conduct a specific study using an eye tracker? One important source is the scientific literature. In the past 50 years, many eye movement researchers have not only published their results, theory, and conclusions, but also published detailed recipes for collecting or analyzing eye tracking data or even made their tools freely available (e.g., Stampe, 1993; Engbert & Kliegl, 2003; Hansen & Pece, 2005; Nyström & Holmqvist, 2010; Hooge & Camps, 2013; Lao et al., 2017; Rousselet et al., 2017; Niehorster et al., 2020a; Niehorster et al., 2023). In this article, we will introduce many eye tracking tools that tackle a wide range of problems. This includes tools for data recording and cleaning, classification of fixations and other events, and to generate areas-of-interest and conduct analyses with them. We will not deliver a ready-to-use pipeline for conducting eye tracking research in this article, since we do not believe there is a one-size-fits-all solution. Nor do we aim to provide an exhaustive list of tools.

The aim of this article is to improve the level of individual eye tracking researchers and their operation. As such, in this article we share our knowledge and experience creating our own tools and using tools created by others. We do not only adopt the perspective of researchers using eye tracking, but also that of supervisors of students using eye tracking, of teachers of eye tracking courses, of collaborators in research projects where we handled the eye tracking, and that of reviewers for eye tracking studies and papers describing eye tracking tools, importantly in journals such as Behavior Research Methods (BRM) and conference venues such as Eye Tracking Research and Applications (ETRA). To meet the goal of improving the level of individual eye tracking researchers and their operation, we will discuss eye tracking tools from multiple perspectives. First, to provide insight into the kinds of eye tracking tools out there, we have developed a taxonomy of tools. Second, we will discuss how to approach recognizing good tools for adoption into one’s eye tracking study. Furthermore, to help researchers find tools that suit their problem, we will provide a survey of tools for a wide range of problems in eye tracking research. This non-exhaustive list is intended to serve as a hub from which researchers can start their search for tools they need. Finally, we provide a discussion of the human side of tool use. This includes both a discussion of choosing tools that fit, and advice for what a researcher can do to expand their solution space when they do not like where they currently find themselves on the spectrum, and, for instance, want to move more towards the DIY side by gaining useful programming skills. While one can chose to read this article all the way through, this article is written in such a way that the reader can also directly skip to the section(s) of their interest.

## Taxonomy of tools

There are many different kinds of tools for conducting eye tracking research. First, there are *software tools*. Software tools for eye tracking research can be subdivided into at least two categories. One category consists of complete ready-to-use software suites for stimulus presentation that allow recording, visualizing and analyzing eye tracking data, such as Tobii Pro Lab, the SR Research Experiment Builder and Data Viewer bundle, iMotions Lab and EyeTrace (Kübler et al., 2015; Otto et al., 2018).

In contrast to software suites that aim to provide a one-stop solution to all one’s research needs stand dedicated software tools that aim to do only one thing and do that well. Many such tools for solving more specific problems in eye tracking research are made available by authors of articles in journals dedicated to research methods, such as Behavior Research Methods and the Journal of Neuroscience Methods, and also more broad journals such as the Journal of Eye Movement Research. Examples of such tools are tools for fixation classification (Nyström & Holmqvist, 2010; Hessels et al., 2017; van Renswoude et al., 2018; see Andersson et al., 2017; and Hooge et al., 2022a, for further algorithms), Area of Interest analyses (Berger et al., 2012; Hessels et al., 2016, 2018a) and the processing of head-worn eye tracker data (Reimer & Sodhi, 2006; Benjamins et al., 2018; Niehorster et al., 2020b, 2023).

There are also tools that have been made available in the form of written *recipes*. Recipes are basically higher-level descriptions (for instance in the form of pseudo-code) of how a problem is solved and can be used as a plan for programmers to implement their own software (for example recipes, see, e.g., Van der Steen & Bruno, 1995, pp. 3461–3462; Goldberg & Kotval, 1999, pp. 637, 639; Hooge & Camps, 2013, pp. 3–4; Niehorster et al., 2015, pp. 13–14; Hessels et al., 2020a, pp. 21–23). While recipes have the potential downside that the tool cannot directly be downloaded and used on one’s gaze data, they have the benefit that they can be integrated into any existing eye tracking data processing pipeline. Another benefit is that the researcher who implements the tool themselves will have a much better understanding of how it works than if they would have just copied a piece of code and used it without delving into its details.

Finally, there are also *hardware tools* such as chinrests (NIMH-NIF, 2019; MetisVidere, 2020), self-built eye trackers (Barsingerhorn et al., 2018; Hosp et al., 2020), and a button box for use with an eye tracker (Niehorster et al., 2020b).

## Recognizing good tools

When building a study workflow, various considerations crop up. These considerations can be both about the tool per se (e.g., has it been validated and is it well documented?) and about one’s context (e.g., does the team possess the needed math or Python programming skills?). Here we will discuss potential indicators one may use to recognize good tools, whereas considerations one may have in choosing tools that fit one’s context will be discussed below when we consider the human side of tool use. Note that while we discuss these two aspects separately, we recognize that considerations about a tool should take one’s context into account. For instance, well-organized and well-documented code and a good readme may be of less importance to an experienced programmer. We also expect that these two sets of considerations will be used in parallel when choosing tools. For instance, regardless of the presence of good example code, a tool may be immediately judged as unsuitable because one does not have the required skills to understand or use it. Finally, one may explicitly decide not to give weight to some considerations. For instance, one may strategically decide to adopt a tool that one currently does not yet have the skills to understand or use because one deems learning to use the tool and associated skills to be a worthwhile long-term investment. Especially at the beginning of an extended research program such as a PhD project, such long-term investments to expand one’s skills might trump short-term opportunism.

Imagine searching for a tool to process eye tracking data, for instance to classify which parts of a recording are fixations. Searching the Internet for a tool that does just that, one finds multiple tools that promise to provide fixations (and other eye movement events) when fed with some gaze position signals. One would probably even find some journal articles that present a tool in detail and that make the software available (e.g., Komogortsev et al., 2010; Nyström & Holmqvist, 2010; Hein & Zangemeister, 2017; Hessels et al., 2017; van Renswoude et al., 2018). However, at first glance, many of these tools may appear to be hard to use. Furthermore, we have had mixed experiences with trying to use tools we found online. For some tools it was easy to get them to do what they promised, while we never managed to get other tools to deliver what they promised. This might have been because the tools did not run, or worse did run but checks on their output revealed that they did not deliver what they claimed. This was, for instance, due to bugs in the tool or due to assumptions made by the tool that did not apply in our intended use case. That raises the question, when examining a tool found on the Internet, how does one judge the quality of such a tool? Here we present some aspects that may inform one’s evaluation of a tool.

When assessing the quality of a tool, one should of course test whether it performs as advertised. Testing may require sufficient openness in the tool to be able to inspect its methods, and a sufficient understanding of the tool’s methods to be able to judge their appropriateness for one’s data and research question. See the section “Choosing tools that fit” below for considerations about the required skills.

Before starting the labor-intensive tool testing phase, one may wish to check for several other indicators of good tools to make a quick selection from the many search results. The following is a list of such indicators that we ourselves use:^2^

- **Validation**: Has the tool been validated in some way, for instance in an accompanying publication in a journal, or in a text on a blog post or in the tool’s manual? Is a procedure implemented to validate updated versions of the tool, or is it validated only sporadically as part of a publication? Has the tool been validated by third parties? Is the validation replicable?
- **Examples**: Does the tool come with example data on which it can be tested, and with example scripts that show how to use it on the example data?
- **Open source**: Is the source code of the tool available?
- **Code documentation**: Is the source code well organized and well documented? For example, are function inputs and outputs clearly described?
- **User documentation**: Did the makers of the tool provide a good readme or manual? Does it have a tutorial or instruction videos? For examples see (Santini et al., 2017a; Chen et al., 2023; Niehorster et al., 2023).
- **Active maintenance**: Is the tool of a kind that needs to be actively maintained? If so, when was it last updated? Were these updates backwards-compatible or did the updates break existing workflows? Is there only a single maintainer or is the tool maintained by a team? Keep in mind that some tools may require little to no maintenance, while others may need frequent updates. For instance, the GUI of a tool may no longer work correctly on newer operating system versions, and a Python tool may crash because a package on which it depends has changed.
- **Popularity**: Does the tool appear to be used a lot? Tools that are used a lot are not only well understood and accepted, but the likelihood that bugs have been spotted in the tool is also larger. It may be hard to judge whether a tool is used a lot, but a possible indicator may be the number of citations a tool receives in the scientific literature. It should be noted that the number of citations does not necessarily reflect how much a tool is used. We, for instance, suspect that Nyström and Holmqvist (2010) and Hayes and Petrov (2016) are cited more frequently than the presented tools are used. The large number of citations but infrequent use we observe in the literature may come about because the terminology used and the principle discussed in these papers are also relevant to cite on their own.
- **Support**: Is there a good infrastructure around the software? For instance, is there an open forum where users can ask questions and exchange tips? Is there a place where bugs can be reported and are these reports responded to by the software’s developers in a timely fashion?
- **Purpose**: What is the use case or purpose of the tool? Which eye tracker or which data formats does the tool support? Who are the intended or prioritized audience of the tool? Such information may indicate whether a particular use case will stay covered in the future or might no longer be maintained or might even be dropped. It also might provide an indication of whether the tool could become burdened with irrelevant functionality in the future.

## Problem domains and available tools

In this section, we will provide a list of tools that address a wide range of problems that may be encountered in studies using eye tracking. We do not aim to be complete in this list, but instead provide a list of tools that we expect to be useful for the majority of readers. We are also aware that any such list quickly becomes out of date. Instead, this section is meant to serve as a hub, providing a sample of what tools are available and thereby a starting point for researchers to find the tools they need, or to understand the lay of the land when they endeavor to develop their own. The tools listed have been categorized by stage in the experimental workflow. That is, we first discuss tools that can be used for recording gaze data (which may include functionality for building experiments), then tools for data visualization and finally tools for data processing and analysis. Besides tools that have functionality specific to a certain stage of the experimental workflow, there are also software suites whose functionality covers multiple stages. We discuss these in a separate section.

We did not test all tools that we refer to, nor did we only include the most widely used tools because we also think it is important to expose the reader to the breadth of tools that is available and thereby help them find tools for themselves, e.g., through knowing what keywords to use when searching. Even though the vast majority of current eye trackers are video-based, many of the techniques used in the below tools are likely agnostic as to the recording technique used to generate the eye movement data. As such, many tools may work equally well for data recorded with a video-based eye tracker as with data from, for instance, an electro-oculography (EOG) or scleral search coil setup. Furthermore, in searching the literature, we found a preponderance of tools for remote eye trackers and less for wearable eye tracking. Since the discussion in this section is limited to available tools, this entails that there will be less focus on wearable eye tracking tools, despite wearable eye tracking being an area that undergoes rapid development and that has its own unique tool needs (Fu et al., 2024). In the following section, we further limit our discussion to tools that are aimed at processing eye tracking data. More general techniques that can solve parts of analysis problems encountered in eye tracking research, such as object recognition and segmentation techniques (e.g., Cheng et al., 2024a; Ravi et al., 2024; Wang & Liao, 2024) or scene reconstruction techniques (e.g., Rublee et al., 2011; Häne et al., 2013; Wang et al., 2023; Avetisyan et al., 2024) but are not aimed at the analysis of eye tracking data per se will not be discussed. Finally, in the overview below, we will only focus on tools. Adjacent topics that may be of interest to eye tracking researchers are discussed in the other parts of the current article series, such as the operationalization of study concepts using eye tracking measurements in part 2 (Hooge et al., in press) and practical considerations such as data quality, form factor, and calibration requirements in part 3 (Nyström et al., in press).

Finally, we will only discuss tools but not provide recommendations regarding which tools the reader should use because we think this is neither possible nor would it be doing the reader a favor. First, we will not provide judgments of whether the tools listed in this section are any good. Determining whether a tool is good is the responsibility of the individual researcher. The section “Recognizing good tools” discusses some signposts for good tools. Second, determining whether a tool is of use for the reader is not possible for us as authors of this article, since we lack knowledge about the reader’s context (e.g., their research question, budget, knowledge and skills, and their interests). As discussed in the section “Choosing tools that fit”, these are all factors that the reader should take into account themselves when deciding what tools to adopt into their research project. Third, the reader, and not the authors of this article, is responsible for their tool choices and for ensuring that they have sufficient skill and understanding of both their problem and potential solutions to make appropriate tool choices. If we were to make specific recommendations in this paper, we would run the risk that we shortcut the reader’s decision process. This could lead them down the wrong path or preclude their discovery that they should invest in their skills to be able to properly address their research problem.

### Software suites

In this section, we discuss software suites – comprehensive tools that often encompass multiple stages of the research process involving eye trackers. Table 1 gives an overview of various software suites for recording and/or analyzing eye tracking data. It is divided into four sections: Software suites by eye tracker manufacturers; other software suites from commercial companies; open-source and commercial suites that are often used for stimulus presentation and data recording; and suites that focus on eye tracking data analysis.

Perusing Table 1, several interesting points can be made. First, users of suites from the first two sections often have a tool on their hands in which they can perform most steps of working with an eye tracker, from data recording to the processing of their data into eye tracking measures. This stands in contrast to users of tools from the third section, which mostly provide only data recording functionality. Users of such tools will have to look to other tools or self-developed scripts for the visualization and processing of eye tracking data. While it is possible to create such scripts in, for instance, MATLAB and Python, and one can thus integrate them with stimulus presentation and data acquisition scripts created using toolboxes such as PsychToolbox (Brainard, 1997; Pelli, 1997; Kleiner et al., 2007) and PsychoPy (Peirce, 2007; Peirce et al., 2019), these toolboxes do not provide such functionality out of the box.

Second, with the exception of PyTrack (Ghose et al., 2020) and eyetrackingR (Dink & Ferguson, 2015), none of the tools listed in the table incorporate functionality for statistical analysis of eye tracking data, such as a comparison of means between two groups or conditions. For such analyses, the user will normally have to export their measures of interest from the software suite and perform further analysis in statistical environments such as SPSS and R. It should be noted that a significant number of tools advertise being able to create export files that can be directly imported into such statistical analysis environments.

Thirdly, not unexpectedly, software suites developed by eye tracker manufacturers only have support for eye trackers from a single brand, whereas the other software suites predominantly have support for eye trackers from multiple manufacturers or their data.

**Table 1:** Software suites and their functionality

Suite	Recording	Importing	Visualizing	Processing/ analysis	Statistical analysis	Multi-brand	Open-source	Interface
Argus Science	$\checkmark$	$\checkmark$	$\checkmark$	$\checkmark$	−	−	−	GUI
GazePoint	$\checkmark$	$\checkmark$	$\checkmark$	$\checkmark$	−	−	−	GUI
ISCAN	$\checkmark$	$\checkmark$	$\checkmark$	$\checkmark$	−	−	−	GUI
Pupil Labs	$\checkmark$	$\checkmark$	$\checkmark$	$\checkmark$	−	−	−	GUI
SMI	$\checkmark$	$\checkmark$	$\checkmark$	$\checkmark$	−	−	−	GUI
SR Research	$\checkmark$	$\checkmark$	$\checkmark$	$\checkmark$	−	−	−	GUI & Python
Tobii	$\checkmark$	$\checkmark$	$\checkmark$	$\checkmark$	−	−	−	GUI & Websocket
Blickshift	$\checkmark$	$\checkmark$	$\checkmark$	$\checkmark$	−	$\checkmark$	−	GUI & Python, R
EyeWorks	$\checkmark$	−	$\checkmark$	$\checkmark$	−	$\checkmark$	−	GUI & SDK
iMotions	$\checkmark$	$\checkmark$	$\checkmark$	$\checkmark$	−	$\checkmark$	−	GUI & UDP/TCP
Mangold	$\checkmark$	$\checkmark$	$\checkmark$	$\checkmark$	−	$\checkmark$	−	GUI
NYAN	$\checkmark$	−	$\checkmark$	$\checkmark$	−	−	−	GUI
Okazolab	$\checkmark$	−	$\checkmark$	$\checkmark$	−	$\checkmark$	−	GUI & Python, C#, Visual Basic
Cedrus	$\checkmark$	−	−	−	−	$\checkmark$	−	GUI
E-Prime	$\checkmark$	−	−	−	−	$\checkmark$	−	GUI & E-Basic
OpenSesame	$\checkmark$	−	−	−	−	$\checkmark$	$\checkmark$	GUI & Python
Presentation	$\checkmark$	−	−	−	−	$\checkmark$	−	GUI & PCL, Python
PsychoPy	$\checkmark$	−	−	−	−	$\checkmark$	$\checkmark$	GUI & Python
PsychToolbox	$\checkmark$	−	−	−	−	$\checkmark$	$\checkmark$	MATLAB
ZING	$\checkmark$	−	$\checkmark$	−	−	$\checkmark$	$\checkmark$	GUI & C#
ETRAN	−	$\checkmark$	$\checkmark$	$\checkmark$	−	$\checkmark$	$\checkmark$	R
EyeFeatures	−	$\checkmark$	$\checkmark$	$\checkmark$	−	$\checkmark$	−	Python
EyeTrace	$\checkmark$	$\checkmark$	$\checkmark$	$\checkmark$	−	$\checkmark$	−	GUI
EyeTrack	$\checkmark$	$\checkmark$	−	$\checkmark$	−	−	$\checkmark$	GUI
eyetrackingR	−	$\checkmark$	−	$\checkmark$	$\checkmark$	$\checkmark$	$\checkmark$	R
ILAB	−	$\checkmark$	$\checkmark$	$\checkmark$	−	$\checkmark$	$\checkmark$	GUI & MATLAB
Gazealytics	−	$\checkmark$	$\checkmark$	$\checkmark$	−	$\checkmark$	$\checkmark$	GUI
GazeAlyze	−	$\checkmark$	$\checkmark$	$\checkmark$	−	$\checkmark$	$\checkmark$	GUI & MATLAB
GazeR	−	$\checkmark$	−	$\checkmark$	−	$\checkmark$	$\checkmark$	R
GraFIX	−	$\checkmark$	−	$\checkmark$	−	$\checkmark$	$\checkmark$	GUI
OGAMA	$\checkmark$	$\checkmark$	$\checkmark$	$\checkmark$	−	$\checkmark$	$\checkmark$	GUI
PerceptionToolkit	−	$\checkmark$	$\checkmark$	$\checkmark$	−	$\checkmark$	$\checkmark$	Python
pymovements	−	$\checkmark$	$\checkmark$	$\checkmark$	−	$\checkmark$	$\checkmark$	Python
PyTrack	−	$\checkmark$	$\checkmark$	$\checkmark$	$\checkmark$	$\checkmark$	$\checkmark$	Python

The columns indicate the following: *Suite*: Short name of the tool. Tools are divided into four sections (tools from eye tracker manufacturers, third party commercial tools, stimulus presentation suites with support for recording eye tracking data, suites for analyzing eye tracking data); *Recording*: Has the ability to record eye tracking data in the program, possibly by means of plugins; *Importing*: Can import recorded data; *Visualizing*: Can create eye tracking data visualizations; *Processing/analysis*: Can process eye tracking data, such as performing fixation classification and AOI analysis or calculation of metrics such as average fixation duration; *Statistical analysis*: Can perform statistical analysis on outcome metrics; *Multi-brand*: has support for eye trackers from more than one brand; *Open source*: Is open source; *Interface*: Interface through which user interacts with the program. Can be a Graphical User Interface (GUI) and/or one or multiple programming languages or communication protocols by means of which functionality of the program can be accessed or extended, or by means of which the program can be remotely controlled.

The short names used in the table refer to the following: *Argus Science* – ETVision and ETAnalysis (https://www.argusscience.com); *Gazepoint* – Gazepoint Analysis (https://www.gazept.com); *ISCAN* – ISCAN DQW, PRZ & GMT (https://www.iscaninc.com); *Pupil Labs* – Pupil-Labs Pupil Invisible/Neon Companion & Pupil Cloud (https://pupil-labs.com); *SMI* – SMI Experiment Center & BeGaze; *SR Research* – SR Research Experiment Builder, Data Viewer & WebLink (https://www.sr-research.com); *Tobii* – Tobii Pro Lab (https://www.tobii.com);

*Blickshift* – Blickshift Recorder & Blickshift Analytics (https://www.blickshift.com); *EyeWorks* (https://www.eyetracking.com); *iMotions* – iMotions Lab (https://imotions.com); *Mangold* – Mangold Vision (https://www.mangold-international.com); *NYAN* – Interactive Minds NYAN (https://www.interactive-minds.com); *Okazolab* – Okazolab EventIDE (https://www.okazolab.com);

*Cedrus* – Cedrus SuperLab (https://cedrus.com); *E-Prime* – Psychology Software Tools E-Prime (https://pstnet.com); *OpenSesame* – Mathôt et al. (2012) (https://osdoc.cogsci.nl); *Presentation* – Neurobehavioral Systems Presentation (https://www.neurobs.com); *PsychoPy* – Peirce (2007); Peirce et al. (2019) (https://www.psychopy.org); *PsychToolbox* – Brainard (1997); Pelli (1997); Kleiner et al. (2007) (http://psychtoolbox.org); *ZING* – ZING (Hosp & Wahl, 2023b) & ZERO (Hosp & Wahl, 2023a) (only for virtual reality);

*ETRAN* – Zhegallo and Marmalyuk (2015, https://github.com/PMarmalyuk/ETRAN/); *EyeFeatures* – https://github.com/hse-scila/EyeFeatures; *EyeTrace* – Kübler et al. (2015), including the experimenter plugin (Otto et al., 2018); *EyeTrack* – EyeTrack, EyeDoctor & EyeDry (Abbott, 2011, see https://websites.umass.edu/eyelab/software/) (only for reading studies); *eyetrackingR* – Dink and Ferguson (2015); *ILAB* – Gitelman (2002); *Gazealytics* – Chen et al. (2023); *GazeAlyze* – Berger et al. (2012); *GazeR* – Geller et al. (2020); *GraFIX* – Saez de Urabain et al. (2015); *OGAMA* – Voßkühler et al. (2008) (http://www.ogama.net); *PerceptionToolkit* – Perception engineer’s toolkit (Kübler, 2020); *pymovements* – Krakowczyk et al. (2023); Jakobi et al. (2024); *PyTrack* – Ghose et al. (2020)

### Data recording

In this section, we will discuss software tools for eye tracking data acquisition.

#### Plugins for stimulus presentation programs

Imagine a face perception researcher who has already set up a successful research line and in that process amassed a range of experiments that were built using a GUI in a stimulus presentation tool like PsychoPy. Now, for a next study, they want to use an experiment paradigm similar to a previous study, but add eye tracking. An obvious route to creating this new experiment is to take a previous experiment and implementation integrate eye tracking into it. Is this possible? Luckily for our face perception researcher, the answer is that they will likely be able to integrate eye tracking into their existing experiment. The stimulus presentation tool they chose, PsychoPy, comes with multiple plugins that enable communication between PsychoPy and a host of eye trackers (PyGaze, Dalmaijer et al., 2014; and the ioHub component of PsychoPy, Peirce et al., 2019). PsychoPy can furthermore be extended to communicate with more eye trackers by using eye tracker control libraries (a collection of functions to control an eye tracker, see below). Researchers who have built their experiments using other tools will likely also be able to record eye tracking data using plugins. Similarly to PsychoPy, OpenSesame (Mathôt et al., 2012) comes with plugins for integrating eye tracking and can be extended using eye tracker control libraries. PsychToolbox comes with the EyelinkToolbox (Cornelissen et al., 2002) and can be extended with several other eye tracker control libraries (e.g., Gibaldi et al., 2017; Niehorster et al., 2020a; Niehorster & Nyström, 2020). The Opticka experiment GUI for PsychToolbox (Andolina, 2024), for instance, has support for several other eye trackers built in. The situation is similar for users of tools such as E-Prime (Psychology Software Tools) and Presentation (Neurobehavioral Systems) in that eye tracker extensions are available for both and that eye tracker control libraries can be used.

When support for a specific brand or model of eye tracker is not available in one’s stimulus presentation program, an extension may be available from the eye tracker manufacturer, or have been posted online by another user of the stimulus presentation program. Using such extensions from sources other than the stimulus presentation program or extending their functionality using eye tracker control libraries may require some programming skills. The required skills may be no more than being able to take example code and adapt it, or to copy-paste code for controlling the eye tracker into some custom code element at the right location in one’s existing experiment.

#### Data recording programs

It is possible to record eye tracking data without presenting a stimulus. Some programs, often those that come with wearable eye trackers such as Tobii Pro Glasses 2 Controller and Pupil Recorder, are made for the sole purpose of recording data. They may run on a standard computer but also on a mobile phone (e.g., the Pupil Neon and Invisible Companion apps, the SMI ETG 2w smart recorder and the Argus Science ETPhone app). Besides such dedicated data recording programs, the stimulus presentation programs mentioned in the previous section as well as manufacturer software can also be used just to record eye tracking data. This may be desirable, for instance, when doing eye tracking studies using world-bound eye trackers without a screen (see, e.g., Gredebäck et al., 2010; Nyström et al., 2017; Thorup et al., 2018; and Valtakari et al., 2021, for a discussion). There are also dedicated tools such as Blickshift Recorder that can be used for this purpose to record from an eye tracker, a scene video and other data streams. Finally, there are tools, such as the lab streaming layer ecosystem (Kothe et al., 2024), for recording and directly streaming eye tracking data over a network connection.

#### Eye tracker control libraries

An eye tracker control library is a collection of programming functions that enable the control of an eye tracker. Controlling an eye tracker entails actions such as performing a calibration and starting or stopping data acquisition. Many eye tracker manufacturers provide such eye tracker control libraries for their products in the form of a software development kit (SDK). These support one or sometimes several popular programming languages, such as C, C#, Python and MATLAB. Readers with programming skills may be able to use SDKs for integrating the eye tracker in the stimulus presentation programs mentioned above, or to develop their own tools for controlling their eye tracker.

The SDKs have also been used to develop convenience packages for using an eye tracker in stimulus presentation environments. Some of these are specific to eye trackers from a given manufacturer (SMI: Niehorster & Nyström, 2020; SR Research: Cornelissen et al., 2002; Tobii: Niehorster et al., 2020a) or even a specific commercial (Tobii Pro Glasses 2: De Tommaso and Wykowska, 2019) or open-source (Sogo, 2017) eye tracker. Other tools are generic interfaces that allow controlling eye tracker systems from several manufacturers using a single interface (Dalmaijer et al., 2014; and the ioHub component in Peirce et al., 2019, for screen-based eye trackers; and Hosp & Wahl, 2023a, for VR). By providing a single uniform interface to a range of eye trackers, such generic tools may not expose all the capabilities of an individual eye tracker to the researcher.

### Data visualization and exploration

Data visualization may be an important part of the research process. Visualizations of the recorded eye tracking data can be useful for, for instance, quality checks, verification that processing steps in the study analysis pipeline (e.g., fixation classification) function as required, to provide insight into the analysis pipeline and make it more explainable, for exploration of participant behavior, or to provide intuitive insight into a study’s findings (see, e.g., Koch et al., 2023). The visualization of eye tracking data is a vast field where many purpose-built techniques (Kurzhals et al., 2017a) have been developed (see Blascheck et al., 2014, 2017, for overviews as well as example visualizations; and Sundstedt & Garro, 2022, for an overview of tools specific to 3D environments) and are evaluated (see, e.g., Claus et al., 2023). Here we will briefly consider tools for common visualizations related to eye tracking. These can be roughly divided into two categories, visualizations that only show the spatial distribution of gaze such as heatmaps (Wooding, 2002b), and visualizations that also contain information about the temporal component of looking behavior, such as scanpaths and scarf plots (Camilli et al., 2008; Wu & Munzner, 2015).

Some visualization tools are dedicated to producing a specific visualization. Examples of dedicated heatmap tools are tools for offline (Wooding, 2002a, b; Špakov & Miniotas, 2007; Stellmach et al., 2010a) or real-time (Duchowski et al., 2012; Pfeiffer & Memili, 2016) generation of heatmaps, eyeScrollR (Larigaldie et al., 2024) for making heatmaps for gaze data recorded on scrolling webpages, for statistical analysis (Caldara & Miellet, 2011; Lao et al., 2017), and the GazeAlyze toolbox (Berger et al., 2012) for generating heatmaps in MATLAB. While a standard heatmap visualization only shows the spatial distribution of gaze, the Heatmap Explorer tool (Tula et al., 2016; see also Wooding, 2002a) allows visualizing the temporal component of gaze data by making it possible to generate heatmaps for specific temporal intervals. Other visualizations that contain information about the temporal component of looking behavior and that can be generated by dedicated tools include scanpaths (Camilli et al., 2008; D’Angelo et al., 2019), time plots (Räihä et al., 2005), arrow plots (Hooge & Camps, 2013), scarf plots (Wu and Munzner, 2015; https://gazeplotter.com), and techniques for producing scale-filtered scanpaths by Rodrigues et al. (2018) and a directional map-based technique (Peysakhovich & Hurter, 2018a) that emphasizes saccade directions in rendered scanpaths.

Instead of being dedicated to a single visualization, most visualization tools support producing a range of different visualizations. Here we will give some examples for different application areas to guide the reader to tools of potential interest. The reader who does not find what they are looking for among these examples or is looking for a more complete overview is referred to Blascheck et al. (2014, 2017), Kurzhals et al. (2017a), and Sundstedt and Garro (2022). Example visualization tools are VERP Explorer (Demiralp et al., 2017) and Gazealytics (Chen et al., 2023) which focus on gaze sequence analysis, frameworks for analysis of eye tracking data captured in VR (Ugwitz et al., 2022) and AR (Pathmanathan et al., 2023; David et al., 2024) environments, the work by Burch et al. (2019, 2021) focused on cartography, by Tang et al. (2012) for reading research, and by Menges et al. (2020) for web stimuli.

### Data processing and analysis

There are GUI applications (e.g., Voßkühler et al., 2008; Kübler et al., 2015) as well as program libraries written in Python (Ghose et al., 2020; Krakowczyk et al., 2023), R (Geller et al., 2020) and MATLAB (Gitelman, 2002) that can perform many of the below processing steps, such as data filtering, fixation classification and AOI analysis. These will not be mentioned repeatedly in the below sections but are discussed in the “Software suites” section above.

#### Data filtering

Many operations that one may apply to eye tracking data may act as a filter. A filter is any manipulation of an (eye tracker) signal that may alter the frequency content of the signal. This article is not about filter theory, for more about that see for instance Proakis and Manolakis (1996). An example of an operation that has filter properties is downsampling. Downsampling reduces the bandwidth of the signal, i.e., it reduces and removes high-frequency aspects of the signal. Filters, depending on their properties, may affect the eye movement properties derived from the signal, such as saccade peak velocity (Juhola, 1986; Mack et al., 2017).

Filtering may be applied at multiple stages during eye tracking data analysis and for different purposes. Here we discuss example motivations to filter eye tracking data, along with references that provide further discussion of these use cases and tools to address them. Besides the aforementioned downsampling, filters are for instance often used to remove unwanted components from the eye tracker signal. Like any signal, eye tracking signals contain not only the behavior of interest but also unwanted variability. Sources of this variability, often called “noise”, include human behavior and measurement noise of the eye tracker (Mack et al., 2017; Niehorster et al., 2020d, 2021). For various uses it is desirable to filter out this noise. For instance, filtering is often used for interactive applications where gaze data is used online (Špakov, 2012; Feit et al., 2017). Removing unwanted components from eye tracking signals requires great care because “denoising [...] is challenging since eye movements are usually non-repetitive making the [...] signal unpredictable.” (Pettersson et al., 2013, p. 2). Many filters have been designed for denoising (or smoothing) the gaze position signal. Some have been designed for online use (e.g., Stampe, 1993; Olsson, 2007; Chartier & Renaud 2008; Toivanen, 2016), and others for offline use (Engelken et al., 1990; Juhola, 1991; Pekkanen & Lappi, 2017; Blignaut, 2019). There are also methods that are specifically aimed at removing sudden large jumps in the gaze signal (Stampe, 1993; Abdulin et al., 2017; see also Chartier & Renaud, 2008).

Besides smoothing data, another common use for filters is to produce velocity and acceleration signals. Velocity signals may, for instance, be used to study saccade dynamics (Tabernero & Artal, 2014; Hooge et al., 2015) or be used as part of an event classification algorithm (e.g., Van der Steen & Bruno, 1995; Engbert & Kliegl, 2003; Nyström & Holmqvist, 2010). The simplest way to compute a velocity signal is to subtract adjacent gaze positions from each other and divide by the inter-sample interval. This works well if the signal has very low noise (high precision), but not if the signal is noisy and measured at a high frequency. In this case, this so-called two-point central difference method has problematic characteristics in that the high-frequency noise in the signal is amplified (e.g., Bahill et al., 1982), potentially obscuring the actual eye movement. The filter properties of different ways to calculate velocity signals and their impact on the resulting estimated eye movement dynamics has received much research (e.g., Bahill et al., 1981; Bahill et al., 1982; Bahill & McDonald, 1983; Jantti et al., 1983; Inchingolo & Spanio, 1985; Juhola, 1986; Mack et al., 2017). Many filters have been designed to suppress the unwanted noise when calculating derivatives of eye tracker signals (Engelken et al., 1982; Inchingolo & Spanio, 1985; Engelken & Stevens, 1990; Das et al., 1996; Nyström & Holmqvist, 2010).

Finally, data loss may occur in eye tracking signals, presenting as gaps in the eye tracking data. Gucciardi et al. (2022) present a filter for the filling of gaps in eye tracking signals, but it should be noted that many event classification algorithms also include procedures for such gap filling (e.g., Hessels et al., 2017; van Renswoude et al., 2018).

#### Converting pixels and millimeters to degrees

Eye tracking measures such as saccade amplitude and gaze direction may be reported by the eye tracker or the tools one uses in various units, such as pixels and millimeters, but are normally reported in degrees (e.g., following the guidelines of Dunn et al., 2023, for reporting eye- and vision-related quantities). If the angle in question is below 10°, then converting from pixels or millimeters to degrees is simple. In this situation, the small angle approximation (Wikipedia, 2024) can be used and the conversion can be done with a constant scaling factor (e.g., the number of pixels per degree) computed for one’s setup. Doing so incurs an error of less than 1% for angles under 10°, and thus works fine if one, for instance, works with a remote eye tracker on a small screen. When working with visual stimuli, it may further be useful to come armed with the rule of thumb that the nail of one’s thumb subtends about 1.5° at arm’s length (O’Shea, 1991), and with the knowledge that 1cm on the screen subtends 1° at an eye-to-screen distance of 57cm. The small angle method applies to converting distances (or sizes) centered on the screen to angles. Gaze positions on a screen are, however, often provided in pixels with an origin in the top-left of the screen. If one wishes to convert such gaze positions to gaze directions in degrees, one additionally needs to make the reported gaze position relative to the center of the screen to be able to apply the small angle method.

When working with extents exceeding 10° such as on bigger screens, or even with wearable eye trackers where gaze shifts of 145° may well be observed (Hooge et al., 2024), the small angle method would lead to huge errors. In this case, proper methods such as trigonometry should be used for converting distances to angles. Hutton (2019) provides an online tool that does the heavy lifting. This tool can also handle converting distances that are not centered on the screen. One complication of working on a flat screen is that the viewing distance to various locations on the screen differs. This is taken into account when using trigonometry, whether through this tool or otherwise. Using the tool by Hutton (2019) however still leaves room for some errors, in that it calculates the horizontal and vertical extent of objects separately. When working with angles instead of Cartesian coordinates, the horizontal and vertical dimensions are no longer independent, which means that the order of the rotations matters. For a full treatment of this problem in eye tracking and correct solutions, the reader is referred to Haslwanter (1995), or to a set of conversion functions that ship with PsychoPy (https://psychopy.org/api/tools/monitorunittools.html).

#### Event classification

Depending on their research question, many researchers are interested in fixations, saccades, blinks and perhaps other events. However, this is not what most eye trackers deliver. Instead, the eye tracking signal consists of a regularly sampled sequence of eye orientations or gaze positions indicating where the participant looked, often at a high sampling frequency. As such, a processing step is needed that applies labels to parts of the eye tracker signal, segmenting the signal into episodes that are meaningful and usable for the researcher. Such methods that process the eye tracking signal into meaningful units such as fixations and saccades are called event classifiers (or event detectors). Classification can, in principle, be performed manually, but this is rarely a good option if algorithmic approaches are available (Hooge et al., 2018). There are a lot of different methods for labeling fixations, saccades and other events in an eye tracker signal (Hein & Zangemeister, 2017, provide a comprehensive overview of methods suitable for data from video-based eye trackers, but also EOG and other eye tracking techniques). These different methods can show marked differences in terms of the number of classified fixations and saccades and their properties (e.g., Andersson et al., 2017; Hessels et al., 2017), although Hooge et al. (2022a) have recently argued these differences may be significantly reduced once one considers adequate post-processing of the classified fixations or saccades. In this section, we will provide a brief overview of various methods for the classification of fixations, saccades and other events in eye tracking data.

##### Fixation and saccade classification

The operationalization of fixation may depend on the frame of reference in which the eye movement data is expressed, and thereby on whether the eye tracker is fixed to the world or to the head. For instance, when an observer sporting a wearable eye tracker walks over a path and fixates a flower along the side of the path, they produce a gaze fixation that is stable in the world with respect to the flower, but an eye orientation that continuously changes with respect to the head as they walk past the flower. Lappi (2016), Hessels et al. (2018b), and Nyström et al. (in press) provide extensive discussions of this point.

We will first consider the classification of fixations and saccades in world-referenced signals, such as gaze position on a screen. Such classifiers for fixations and saccades can be divided into two categories, so-called fixation pickers and saccade pickers (Karn, 2000). Which type of classifier one may wish to use depends on whether one is interested primarily in fixations, or in saccades. Fixation pickers focus on labeling fixations, and employ explicit rules for determining which segments of an eye tracker signal meet the criteria of a fixation (e.g., the recorded gaze positions are within a certain distance from each other for a certain minimum time). Saccade pickers on the other hand have explicit rules for labeling saccades (e.g., eye velocity exceeds a certain minimum speed). Methods for classifying saccades in eye tracking signals have been around for a long time (e.g., Baloh et al., 1980; Juhola et al., 1986; Van der Steen & Bruno, 1995), including even methods that are locally adaptive to the noise in the eye tracking signal (Tole & Young, 1981; Juhola et al., 1985; see also, e.g., Engbert & Kliegl, 2003; Nyström & Holmqvist, 2010; Mould et al., 2012). There are saccade classifiers that focus on specific use cases, such as detecting saccades during smooth pursuit (Sauter et al., 1991; Liston et al., 2013; Daye & Optican, 2014; Niehorster et al., 2015), for online detection of saccades (Han et al., 2013; Schweitzer & Rolfs, 2020; Elmadjian et al., 2023) and methods specialized for microsaccades (Engbert & Kliegl, 2003; Bettenbühl et al., 2010; Mihali et al., 2017), or for VR environments (Diaz et al., 2013). Various fixation classifiers have also been introduced (e.g., Wijnen & Groot, 1984; Salvucci & Goldberg, 2000; Blignaut, 2009; Komogortsev et al., 2010; Olsen, 2012; Krassanakis et al., 2014), including some that are designed to provide robust classification of low quality data such as observed in infant participants (Hessels et al., 2017; van Renswoude et al., 2018), or that are specialized for data recorded in VR environments (Weber et al., 2018; Llanes-Jurado et al., 2020). Implementations of a selection of fixation and saccade classification algorithms and common post-processing routines are available from Hooge et al. (2022a).

There are also dedicated algorithms for classifying eye movements in data from wearable eye trackers. Examples of such algorithms are (Reimer & Sodhi, 2006; Larsson et al., 2016; Hessels et al., 2020b; Kothari et al., 2020). This is an area of active research (e.g., Kinsman et al., 2012; Drews & Dierkes, 2024).

##### Classification of other events

Besides algorithms focused on classifying fixation or saccades, there are also algorithms that classify other types of events, such as smooth pursuit and post-saccadic oscillations (PSO). These algorithms often classify these events alongside fixations and saccades. For instance, Komogortsev and Karpov (2013), Larsson et al. (2015), Pekkanen and Lappi (2017), Startsev et al. (2019), and Dar et al. (2021) additionally classify pursuit, and Larsson et al. (2013) and Zemblys et al. (2018, 2019) PSO. Santini et al. (2016) and Elmadjian et al. (2023) provide online classifiers for fixations, saccades and pursuit.

##### Blink classification

Besides the above events that describe movement of the eye or lack thereof, one may also wish to classify blinks, i.e., motions of the eyelid. Blink classification can for instance be used when blinks themselves are of interest (see Nyström et al., 2024) or used to separate data loss due to blinks from other causes. There are various methods for doing so, based on a video of the eye (e.g., Sanchis-Jurado et al., 2020), on the pupil signal of an eye tracker (Pedrotti et al., 2011; Mathôt, 2013; Hershman et al., 2018), and on an eye openness signal provided by some eye trackers (Nyström et al., 2024).

##### Using an algorithm

What technical problems may one encounter when trying to use a classifier found online? One problem is that the data provided by the eye tracker does not match the expected input format for the classifier. In this case, a data input function has to be written to solve issues such as ensuring that timestamps have the right unit, that gaze data columns have the expected names and that gaze data are converted to degrees (see the section “Converting pixels and millimeters to degrees”) if the classifier requires that but the gaze data provided by the eye tracker is expressed in pixels on a computer screen. Another consideration might be how to evaluate the performance of event classification algorithms. Startsev and Zemblys (2023) provides a discussion of this problem.

#### Area of interest analysis

One common use of eye trackers is to answer questions about what participants look at. The analysis approach for answering such a question often involves areas of interest (AOIs, also known as regions of interest [ROIs], or interest areas [IAs]). AOIs are regions in the visual stimulus delineating where, e.g., objects of interest are located. When performing an AOI analysis, gaze data are tested for whether they fall on the AOI (AOI hits). This enables determining what objects or areas are looked at, but also other aspects of spatiotemporal looking behavior such as when objects are looked at and for how long (Goldberg & Helfman, 2010a).

What methods and tools are there for creating AOIs and using AOIs to analyze gaze data of participants looking at static stimuli (e.g., images)? Many of the software suites discussed above support AOI analysis based on hand-drawn AOIs, for instance using shapes such as rectangles, ovals and polygons. The Titta toolbox for Tobii eye trackers (Niehorster et al., 2020a) provides examples in MATLAB and Python for performing AOI analysis using binary AOI mask images that can be created in any photo editing software. Besides these approaches, tools have been published for creating AOIs that are robust to offsets in eye tracking data (Hessels et al., 2016), and for creating AOIs based on salience modeling of visual stimuli (Privitera & Stark, 2000; Fuhl et al., 2018b) or on the spatial distribution of gaze data (Santella & DeCarlo, 2004; Fuhl et al., 2018a). Nuthmann et al. (2017) provide a method and toolbox for using gridded AOIs, which also provides for statistical evaluation of such data. Finally, Rim et al. (2021) introduced an alternative method that avoids having to delineate AOIs but instead uses points of interest.

What to do with dynamic stimuli (e.g., videos and animations) where AOIs are not stationary over time? Here we discuss the case for screen-based eye tracking studies. AOI tools for a specific type of dynamic stimulus, those created by the participant themselves with the scene camera of a wearable eye tracker, are discussed in the wearable eye tracking section below. Various software suites support AOI analyses on videos and other dynamic stimuli. A common method implemented in such software is for researchers to define AOIs on key frames, after which the software determines the AOI position and shape on intermediate frames using interpolation. There are also dedicated tools for AOI analysis on dynamic stimuli. Papenmeier and Huff (2010) provide an automated method for AOI analysis in dynamic computer-generated scenes. Their method can, however, be adapted for use with real-world videos if a 3D reconstruction of the scene depicted in the video is available. Several other methods are available for determining what object is looked at in computer-generated 3D (Holmberg, 2007; Stellmach et al., 2010b; Sundstedt et al., 2013; Bernhard et al., 2014) or 2D (Alam & Jianu, 2017; Jianu & Alam, 2018) environments, for automatic construction of AOIs for faces in eye tracking setups for interpersonal interaction (Hessels et al., 2018a; Vehlen et al., 2022), and for tracking manually annotated (Bonikowski et al., 2021) or automatically detected (Kang et al., 2016) AOIs across video frames. Finally, a method has been developed for visually performing AOI analyses using space-time cubes (Kurzhals et al., 2014).

#### Higher-order measures

From the output of an AOI analysis or fixation classification, higher-order descriptions of spatiotemporal looking behavior can be determined. One common representation of looking behavior is scanpaths, the sequential pattern of fixations. Scanpaths provide information about, for instance, the visual strategy when performing a task (Noton & Stark, 1971; Ballard et al., 1995) and are frequently used for comparing or classifying strategies used by participants or participant groups.

To perform such analyses, there are various scanpath analysis techniques (Eraslan et al., 2015; see Anderson et al., 2015; French et al., 2017; Dewhurst et al., 2018; Fahimi & Bruce, 2021, for evaluations), such as scanpath comparison methods (e.g., Privitera & Stark, 2000; Josephson & Holmes, 2002; West et al., 2006; Cristino et al., 2010; Duchowski et al., 2010; Dewhurst et al., 2012; de Bruin et al., 2013; Le Meur & Baccino, 2013; Dolezalova & Popelka, 2016; Peysakhovich & Hurter, 2018b; Newport et al., 2022; and an R implementation of Dewhurst et al., 2012, https://github.com/bbuchsbaum/eyesim), including methods for dynamic stimuli (Dorr et al., 2010; Salas & Levin, 2022); methods for simplifying scanpaths or identifying average scanpaths (Goldberg & Helfman, 2010b; Grindinger et al., 2010; Eraslan et al., 2016, 2018); and methods for classifying scanpaths (e.g., Haass et al., 2016; Kübler et al., 2017; Coutrot et al., 2018; Fuhl et al., 2019a; b; Kucharský et al., 2020), including methods for dynamic stimuli (Grindinger et al., 2011) and specific to reading (von der Malsburg & Vasishth, 2011; Ma et al., 2023).

Gaze behavior can also be represented as a sequence of transitions between different AOIs. For some analyses, the transitions themselves are of interest. There are tools that focus specifically on transition matrices (Goldberg & Kotval, 1999; Hooge & Camps, 2013; Chen et al., 2023). Measures of the degree of orderliness of gaze behavior can be computed from the scanpath or the transition matrix (Ellis & Stark, 1986; Weiss et al., 1989; Krejtz et al., 2014, 2015; see, e.g., Allsop & Gray, 2014; Niehorster et al., 2019, for applications).

Whereas scanpath measures emphasize the spatial aspects of looking behavior, recurrence quantification analysis instead more directly taps into the temporal aspects of gaze behavior, such as the proportions and temporal dynamics of refixations. Anderson et al. (2013) provide a tool that can perform recurrence quantification analysis (see also Vaidyanathan et al., 2014; and see Gurtner et al., 2019, for an application). Such analysis techniques can also be employed to investigate the interplay in gaze behavior between two participants (e.g., Richardson & Dale, 2005; Jermann et al., 2011; Schneider et al., 2016) through cross recurrence analysis (Dale et al., 2011; Coco & Dale, 2014). Topological tools have also been proposed for these purposes (Hein & Zangemeister, 2017; see also Kasprowski & Harezlak, 2017).

### Special topics

In this section we will discuss specialized tools that are designed for the study of a specific topic using eye tracking (e.g., reading) or that support a processing step or the use of a specific technique.

#### Gaze estimation, calibration, and offset correction

Gaze estimation is the process of mapping raw acquired measures (such as the raw image or the positions of features in the eye image) to eye orientations in the head or gaze positions in the world (e.g., on a screen). To ensure the accuracy of this mapping, it is necessary to perform a user calibration,^3^ through which it is possible to optimize the parameters of the gaze estimation model to the individual. Several articles discuss options for calibrating eye trackers and provide advice on how to perform the calibration, such as how many calibration points to use, how to instruct the participant and how to deal with special populations (Stampe, 1993; Sasson & Elison, 2012; Karl et al., 2020; Hopper et al., 2021; Leppänen et al., 2022; Park et al., 2023; Fu et al., 2024; Niehorster et al., 2024; Zeng et al., 2024; Nyström et al., in press). The reader is referred to these articles for further discussion of these topics.

There are a wide range of approaches to performing gaze estimation based on data acquired with a video camera, see for instance Hansen and Ji (2010) and Liu et al. (2022) for overviews of methods, although there are also methods to, for instance, acquire gaze signals free of baseline drift with EOG eye trackers (Barbara et al., 2024). Gaze estimation can for instance be performed using linear interpolation (Kliegl & Olson, 1981; McConkie, 1981) or through the fitting of various polynomials (e.g., Wijnen & Groot, 1984; Cerrolaza et al., 2012; Blignaut, 2013; Blignaut & Wium, 2013; Lara-Alvarez & Gonzalez-Herrera, 2020; Narcizo et al., 2021), sometimes with additional corner correction factors (e.g., Sheena & Borah, 1981; Stampe, 1993) or through geometric transformations (e.g., Yoo & Chung, 2005; Hansen et al., 2010; Ma et al., 2015; Morimoto et al., 2020). Gaze estimation may also involve geometric eye models that can be used in eye tracking setups using multiple corneal reflections and/or cameras (e.g., Shih et al., 2000; Coutinho & Morimoto, 2006; Guestrin & Eizenman, 2006; Villanueva & Cabeza, 2007; Barsingerhorn et al., 2017) or methods for recovering the visual axis from images of the pupil (e.g., Świrski & Dodgson, 2013; Dierkes et al., 2018; Santini et al., 2019; Su et al., 2020).

Calibration of such gaze estimation methods normally involves asking participant to look at one or multiple fixation targets. There are also various specialist tools, such as methods for collecting data for calibrating head-worn eye trackers based on VOR (Santini et al., 2017b) and by looking at fingertips (Bâce et al., 2018), smooth pursuit-based techniques (O’Regan, 1978; Pfeuffer et al., 2013; Blignaut, 2017; Drewes et al., 2019; Hassoumi et al., 2019), methods for calibrating nystagmus patients (Rosengren et al., 2020), several methods exploiting scene salience or visual task structure for implicit calibration (see Zhang et al., 2024, for an example and overview) and elaborate schemes for calibrating the eye tracker for robust gaze estimation at different pupil sizes (Drewes et al., 2012, 2014; Hooge et al., in press).

Besides tools for the calibration of eye tracking data, there are also tools for reduction of offsets in already calibrated data. Some of these are general methods (e.g., Zhang & Hornof, 2011; Blignaut et al., 2014; Zhang & Hornof, 2014; Vadillo et al., 2015; Blignaut, 2016; Lander et al., 2016; Ramirez Gomez & Gellersen, 2018), while others are designed for specific problems, such as assigning of gaze data to text lines in reading research (e.g., Špakov et al., 2019; Carr et al., 2022; Mercier et al., 2024, see the section on reading for more detail) or improving the accuracy of determining the binocular gaze point in 3D (Essig et al., 2006).

#### Reading

There is a long history of using eye trackers for studying reading (see, e.g., Rayner, 1998; Radach & Kennedy, 2004; Liversedge et al., 2022, for overviews). As such, over the years, specific tools have been developed to support reading research using eye trackers, targeting both the creation of experiments and data recording, and the analysis of eye tracking data during reading. Besides these specific tools, manufacturer software such as provided by SMI, SR Research and Tobii has specific support for the analysis of eye tracking data collected from participants reading sentences or paragraphs, and the ability to compute gaze metrics that are specific to the reading field (see Inhoff & Radach, 1998). These software suites can for instance automatically make AOIs for the words in the text. It is our impression that especially the SR Research Experiment Builder and Data Viewer software bundle is a popular tool among reading researchers. EyeMap (Tang et al., 2012; see also Hegarty-Kelly, 2020) is an open-source tool for the analysis of reading data, and the EyeTrack, EyeDoctor and EyeDry tools from the university of Amherst (Abbott, 2011, see https://websites.umass.edu/eyelab/software/) offer an environment for conducting measurements, visually inspecting and analyzing reading data. Furthermore, there is the popEye toolbox for the R environment (Schroeder, 2019, 2022) and eyekit for Python (Carr, 2023). The cleaning of fixation data for reading analyses, such as removing fixations that are too short, is examined by Eskenazi (2024). Tools for comparing scanpaths during reading are available (von der Malsburg & Vasishth, 2011; Ma et al., 2023).

A common problem encountered in reading studies is that the gaze data is offset from the text stimulus due to inaccuracy of the eye tracking data. Several tools (e.g., EyeMap, Tang et al., 2012; and EyeDoctor, Abbott, 2011) enable manually moving the classified fixations to correct for these offsets (performed by, e.g., Scherr et al., 2016; Vasilev et al., 2021; Adedeji et al., 2024; according to Mercier et al., 2024). However, since the manual correction process is both time-consuming and subjective, it may be preferrable to use automated tools instead (Cohen, 2013). Several such tools are available (e.g., Cohen, 2013; Špakov et al., 2019; Glandorf & Schroeder, 2021; Carr et al., 2022; Mercier et al., 2024). The alignment methods by Glandorf and Schroeder (2021) and Carr et al. (2022) are also implemented in the popEye (Schroeder, 2019, 2022) and eyekit (Carr, 2023) toolboxes.

Besides tools for studying the reading of normal sentences and paragraphs using an eye tracker, there are also tools for studying the reading of computer source code (Guarnera et al., 2018; Behler et al., 2023a, b; Saranpää et al., 2023; Behler et al., 2024; Stolp et al., 2024; Tang et al., 2024), the production of text (Alamargot et al., 2006; Andersson et al., 2006; Wengelin et al., 2009; Chukharev-Hudilainen et al., 2019; Wengelin et al., 2019, 2024), and the translation of text (Carl, 2012; Jakobsen, 2019).

#### Wearable eye tracking

Studies using wearable eye tracking face unique challenges, see for instance Fu et al. (2024) for an extensive discussion and practical advice for setting up studies using wearable eye tracking (see also Nyström et al., in press, for considerations when considering adopting wearable eye tracking in a study). As discussed in several articles (Lappi, 2016; Hessels et al., 2018b; Nyström et al., in press), head-worn eye trackers record eye movements with respect to the head of the participant, not gaze with respect to locations in the world. Researchers are on the other hand most often interested in what or where in the world participants look at. As such, eye movement data collected with a wearable eye tracker needs to be transformed to gaze in the world. Such processing of the recorded data can be done manually (see, e.g., Gidlöf et al., 2013; Häggström et al., 2015; Vansteenkiste et al., 2015; Gidlöf et al., 2017), but this is a subjective and very time-consuming process.

There are many tools and methods that use the gaze data from a mobile eye tracker along with the scene video (a video acquired with a camera that is attached to the participant’s head) to enable determining what is looked at or where in the world the participant looks. These methods include ones based on 3D reconstructions of the scene that gaze can be mapped to directly (Paletta et al., 2013; Booth et al., 2014; Jensen et al., 2017; Singh et al., 2018; Jogeshwar & Pelz, 2021; Stein et al., 2023), or where the reconstructions are annotated by hand (Kopácsi et al., 2023); object recognition and segmentation of the scene video (Wolf et al., 2018; Panetta et al., 2020; Deane et al., 2023; Alinaghi et al., 2024) including methods focused on bodies and faces (Callemein et al., 2019; Hessels et al., 2020a; Jongerius et al., 2021); manual annotation of key frames (Essig et al., 2011; Meyer et al., 2021) or creation of AOI template images (Weibel et al., 2012; Ryabinin et al., 2022; Zhang et al., 2022) which are then tracked throughout the recording; clustering (Kurzhals et al., 2017b), matching (Brône et al., 2011; Toyama et al., 2012), or classification (Panetta et al., 2019; Barz et al., 2023) based on image content around the fixation point in the scene video; or AOI placement based on fiducial markers visible in the scene video (Weibel et al., 2012; Kiefer et al., 2014; Pfeiffer et al., 2016; Duchowski et al., 2020; Tabuchi & Hirotomi, 2022). There are also methods that specialize in mapping gaze to one or more screens in the environment, either using fiducial markers (Faraji et al., 2023) or based on only the scene camera video (Mardanbegi & Hansen, 2011; Turner et al., 2012; Paletta et al., 2014a, b; Lander et al., 2015; Batliner et al., 2020). Lastly, there is a tool using a poster with fiducial markers for automated data quality (accuracy and precision) assessment for mobile eye tracking data (Niehorster et al., 2023).

Other methods for mapping gaze to the world use additional sensors, such as motion capture (Allison et al., 1996; Johnson et al., 2007; Ronsse et al., 2007; Herholz et al., 2008; Essig et al., 2012; Pfeiffer, 2012; Cesqui et al., 2013; Burger et al., 2018; Langstrand et al., 2018; Lavoie et al., 2018; Narasappa, 2022; Stone et al., 2024), fiducial marker-based head tracking systems (Hooge et al., 2024) or inertial measurement units (IMUs, Larsson et al., 2016; Tomasi et al., 2016; Matthis et al., 2018).

Finally, there are some open tools for the control and data acquisition of wearable eye trackers (De Tommaso & Wykowska, 2019; Nasrabadi & Alonso, 2022), and for the analysis of such data (Benjamins et al., 2018; Niehorster et al., 2020b).

#### Gaze-contingent displays

Gaze-contingent manipulations are techniques where the stimuli depend on data from an eye tracker. They may be as simple as starting the trial when the participant looks at a fixation cross, can involve altering what stimuli are shown to the observer based on where the observer is looking, and may even involve changing the content of the display during saccades. Gaze-contingent techniques have been used to effect several manipulations of visual stimuli, and have notably been used in research where only part of the display near where one looks is visible during reading (McConkie & Rayner, 1975; Rayner, 2014) and scene viewing (van Diepen et al., 1998; Nuthmann, 2014; Nuthmann & Canas-Bajo, 2022), research using artificial scotomas where foveal vision is blocked (Rayner & Bertera, 1979; Cornelissen et al., 2005; Biebl et al., 2022) or degraded (Jordan et al., 2012; Cajar et al., 2016), research into multiresolution display techniques such as foveated rendering where the rendered level of detail depends on where the observer looks (see Mohanto et al., 2022, for a review), studies using gaze-contingent displays for gaze guidance (e.g., Barth et al., 2006; Bailey et al., 2009; Sridharan et al., 2015) and studies where the content of the screen is manipulated while saccades are in flight to study, for instance, motor learning (McLaughlin, 1967; Frens & van Opstal, 1994; Havermann et al., 2011). Furthermore, there are even studies with young children that have used techniques where the behavior of an on-screen avatar depends on where the children look to study social perception (Vernetti et al., 2017, 2018; Wang et al., 2020) and learning (Tsuji et al., 2021), studies where gaze-contingent displays are used for rehabilitation of brain disorders (see Carelli et al., 2022, for a review) or other therapeutic efforts (Machner et al., 2020) and studies where the display content was contingent on the behavior of one or multiple other participants (Brennan et al., 2008; Niehorster et al., 2019; Siirtola et al., 2019). Gaze-contingent displays where stimuli are manipulated based on the participant’s eye movements often have strict requirements regarding timing, which requires an eye tracker that can deliver gaze position samples with low latency, fast software for updating the stimulus and a fast graphics display system. Studies such as Loschky and Wolverton (2007) examine the timing requirements for gaze-contingent manipulations.

We have found that many studies used their own custom implementation to create the gaze-contingent manipulation. There are, however, some more general tools, including a hardware tool for low-latency updating of stimulus displays using 60 Hz projectors (Richlan et al., 2013), two combined hardware and software platforms (Reder, 1973; Santini et al., 2007; see McConkie, 1997, for context on Reder’s work), a self-contained stimulus presentation tool designed for presenting gaze-contingently masked stimuli (Orlov & Bednarik, 2016), and two tools for gaze-contingent localized blurring of stimuli (Perry & Geisler, 2002; Malkin et al., 2020). Furthermore, SR Research’ Experiment Builder provides the ability to create gaze-contingent displays, and other software suites such as SMI Experiment Builder and Tobii Pro Lab support to start or stop trials based on the participant’s gaze position. Several other software tools come with gaze-contingent example code for MATLAB (Cornelissen et al., 2002; Niehorster et al., 2020a) and Python (Peirce, 2007; Dalmaijer et al., 2014; Nyström et al., 2017; Niehorster et al., 2020a). Finally, McConkie et al. (1984) and Aguilar and Castet (2011) provide advice for implementing gaze-contingent display paradigms.

### Hardware

In this section, we will discuss various hardware tools, ranging from self-built eye trackers and tools for validating eye trackers, to important aspects of an eye tracker setup that often do not receive sufficient consideration, such as the furniture and design of a lab room.

#### Self-built and webcam eye trackers

Many different self-built or open-source eye trackers can be found in the literature. Here we provide a small sample. Firstly, there are many complete desktop eye tracking systems that are designed to use the pupil and/or corneal reflection(s) (e.g., Ebisawa & Fukumoto, 2013; Sogo, 2013; Balthasar et al., 2016; Wyder & Cattin, 2016; Zimmermann et al., 2016, see https://www.myeyetracker.com; Matsuda et al., 2017; Barsingerhorn et al., 2018; Hosp et al., 2020; Ivanchenko et al., 2021) or the dual Purkinje principle (Chamberlain, 1996; Wu et al., 2023) for gaze estimation. There are also open head-worn eye tracker designs and systems (e.g., Babcock & Pelz, 2004; Li et al., 2006; Rantanen et al., 2011; Mantiuk et al., 2012; Lukander et al., 2013; Kassner et al., 2014; Lanata et al., 2015; Eivazi et al., 2018; Lander et al., 2018; Krohn et al., 2020; Nourrit et al., 2021; Yang et al., 2023). Furthermore, there are system designs focused on pupillometry (Zandi et al., 2021; Martin et al., 2022; Barry & Wang, 2023), VR (https://github.com/EyeTrackVR/EyeTrackVR) and even eye trackers that can be embedded in surgical microscopes (Charlier et al., 1991; Eivazi et al., 2016; Eivazi & Maurer, 2018) and gun scopes (Hansen et al., 2019).

Besides such complete systems, there are also software libraries that provide various implementations of eye tracking algorithms for desktop (Agustin et al., 2010; Casas & Chandrasekaran, 2019; Sadeghi et al., 2024) and head-worn (Santini et al., 2017a) eye tracking, pupillometry (Mazziotti et al., 2021), as well as even software libraries and methods for eye tracking from retinal images (Mulligan, 1997; Stevenson et al., 2010; Bedggood & Metha, 2017; Agaoglu et al., 2018; Zhang et al., 2021).

Although webcam eye tracking has a long history (e.g., Hansen et al., 2002; Hansen & Pece, 2005), in the last decade, the use of standard webcams and smartphone cameras for eye tracking has increased rapidly. This development is boosted by researchers looking to bring experiments to people at home instead of bringing people to dedicated lab facilities. As such, significant research has been done to evaluate the performance of webcam and smartphone-based eye tracking (e.g., Semmelmann & Weigelt, 2018; Yang & Krajbich, 2021; Bánki et al., 2022; Kaduk et al., 2023; Saxena et al., 2023; Van der Cruyssen et al., 2023; Bogdan et al., 2024; Falch & Lohan, 2024; Hagihara et al., 2024; Prystauka et al., 2024; Steffan et al., 2024; Valtakari et al., 2024). Some example tools for eye tracking using a webcam or smartphone are Papoutsaki et al. (2016), Valliappan et al. (2020), Erel et al. (2023), Werchan et al. (2023), https://github.com/NativeSensors/EyeGestures, as well as online tools such as GazeRecorder (https://gazerecorder.com), iMotions WebET (https://imotions.com), SeeSo (https://visual.camp) and LabVanced (https://www.labvanced.com, Finger et al., 2017), but the reader is recommended to consult the overviews of Heck et al. (2023) and Cheng et al. (2024b) if such techniques and tools are of interest.

#### Synchronization and setup validation

Eye trackers may be used in conjunction with other sensors, such as motion capture systems and EEG. Analyzing such multimodal data requires synchronization of data streams from the various sensors. How can this be achieved? Many software suites from eye tracker manufacturers, as well as other companies (e.g., Blickshift, iMotions, Neurobehavioral Systems Presentation) advertise support for synchronized co-recording from an eye tracker and other sensors. There are also hardware solutions for synchronizing displays and various other sensors, such as offered by the VPixx ecosystem, the Cedrus Stimtracker and the Cambridge Research Systems Bits#. Besides these commercial products, there are general open-source solutions that offer support for a wide array of sensors, such as OpenSync (Razavi et al., 2022) and other solutions built upon the LabStreamingLayer (Kothe et al., 2024) infrastructure (see Table 1 in Razavi et al., 2022, for an overview).

Furthermore, there are many tools that enable the synchronization of eye tracking data with data collected from a specific sensor, such as for EEG (Dimigen et al., 2011; Bœkgaard et al., 2014; Ionescu et al., 2022), including in virtual reality setups (Larsen et al., 2024; Salehi et al., 2024); motion capture (Huang et al., 2004; Essig et al., 2012; Cesqui et al., 2013; Burger et al., 2018; Lavoie et al., 2018; Narasappa, 2022; Stone et al., 2024); MRI (Lawrence et al., 2011; Hanke et al., 2020); and audio (Arslan Aydin et al., 2018; Boulay et al., 2023). Other techniques are designed for ensuring or verifying synchronization of the eye tracking data to stimulus presentation systems (Saunders & Woods, 2014; Brooks et al., 2019; Watson et al., 2019), including VR and simulator systems (Medenica & Kun, 2012; Diaz et al., 2013; Loeb et al., 2016). Several products are available to verify the timing of such setups, such as the Blackbox Toolkit and the Neurobehavioral Systems LabStreamer.

It may also be of interest to synchronize multiple eye trackers with each other, whether for studying human behavior (e.g., Hessels et al., 2019; Niehorster et al., 2019; Hessels et al., 2023; Saxena & Fink, 2023) or for the evaluation of eye tracker performance by comparing eye trackers using simultaneous co-recording (e.g., van der Geest & Frens, 2002; Houben et al., 2006; McCamy et al., 2013; McCamy et al., 2015; Titz et al., 2018; Ehinger et al., 2019; Holmqvist et al., 2020; Stein et al., 2021; Aziz et al., 2022) or comparing eye trackers and webcams (e.g., Kaduk et al., 2023; Falch & Lohan, 2024).

It may also be desirable to validate the performance of the eye tracker itself (not to be confused with validation of the eye tracker’s user-calibration). One method for evaluating aspects of the performance of eye trackers is to use artificial eyes (e.g., Crane & Steele, 1985; Hayes & Petrov, 2016; Wang et al., 2017; Ooms & Krassanakis, 2018; Wyder & Cattin, 2018; Holmqvist & Blignaut, 2020; Holmqvist et al., 2021; Niehorster et al., 2021). Many different devices have been designed that are able to move artificial eyes in precisely known ways, so that eye tracker data can be evaluated with a known input (e.g., Abramov & Harris, 1984; Biamino et al., 2005; Bassett et al., 2010; Reingold, 2014; Tannfelt Wu, 2018; Wyder & Cattin, 2018; Holmqvist & Blignaut, 2020; Felßberg & Strazdas, 2022; Sueishi et al., 2022; Lotze et al., 2024).

#### The forgotten parts of an eye tracking setup

When building an eye tracking setup, the focus may be on the eye tracker, the screen and possibly the computer that they are connected to. In our experience, other critical components such as the chin rest, the table, the chair and room lighting might not be considered at all or at best be an afterthought. We are not aware of literature that systematically investigates the impact of choosing a stable table and a chair that keeps the participant from moving on the quality of the recorded eye movement data. From personal experience, we know that these are important aspects of an eye tracking setup that require careful attention.

##### Chinrests and tables

When using an eye tracker that is fixed in the world (see Valtakari et al., 2021; Nyström et al., in press), one may want to consider how to keep participants from moving too much. Even for eye trackers that can ostensibly deal with significant movement of the participant, such movement may lead to large offsets in the gaze data or significant data loss (Blignaut & Wium, 2014; Hessels et al., 2015; Niehorster et al., 2018, for world-fixed eye trackers; see Niehorster et al., 2020c; Hooge et al., 2022b; Onkhar et al., 2023; Velisar & Shanidze, 2024, for wearable eye trackers). If lower data quality is problematic for a study, we recommend that participants are placed on a chin- and forehead rest when possible (see NIMH-NIF, 2019; MetisVidere, 2020, for open-source chinrest designs; and Nyström et al., in press, for considerations regarding the use of chinrests). Furthermore, placing the eye tracker and the chinrest on a sturdy table (i.e., ideally a table with four stable legs) helps to minimize movement of the participant with respect to the eye tracker. Real perfectionists may consider to put the chinrest, mouse, keyboard and other parts of the setup that are in contact with the participant on another table than the eye tracker so that any actions of the participant do not cause the eye tracker to move. Besides physical chinrests, virtual chinrests that keep the participant in place based on visual feedback have been developed (Li et al., 2020) and are available in online experiment environments such as LabVanced (see Kaduk et al., 2023) and jsPsych.

##### Chairs

What happens when participants are put in a chair that can roll, swivel, and pivot, such as a standard desk chair? Participants may use the mobility they are afforded. Such movement may negatively impact the quality of the eye tracker signal. Thus, it makes sense to use chairs that do not afford more freedom of movement than needed for the setup. For instance, instead of using height-adjustable chairs that often have embedded springs, one can use a fixed chair and use a height-adjustable table (e.g., Niehorster et al., 2024). If participants may be very large or small, having extra chairs to accommodate their size may be useful. The freedom to roll, pivot or swivel the chair is often not needed and using a fixed chair reduces the ability of the participant to move around. Standard desk chairs do provide such extra freedom of movement and are therefore not suitable. Besides stable chairs, sometimes neckrests or pillows are used to further fixate participants or increase their comfort (e.g., Trojano et al., 2012; Sprenger et al., 2013; Choe et al., 2016). We know of a further interesting solution to stimulate participants to sit still. Although they did not report it in the paper, Holleman et al. (2023) had children (8–10 years olds) wear a vest with Velcro attached to the back and to the backrest of the chair. Using this method, children felt resistance on their back when they moved, encouraging them to sit still. For younger participants (infants and toddlers), setups using car seats have been developed with the aim to afford them as little movement as possible (see, e.g., Hessels & Hooge, 2019, Figure 2 and associated text for an in-depth discussion).

##### Climate control

If one has equipment that produces significant heat in a small experiment room, such as one or multiple powerful computers, this room may get uncomfortably warm (e.g., Niehorster et al., 2024). This may especially be a problem in the summer months depending on the climate where the lab room is situated. If this is a potential concern, it should be considered whether the lab room has adequate climate control.

##### Room lighting

Finally, depending on the eye tracker and the research question, adequate control over the lighting conditions in the room may be crucial. For some eye trackers, one may wish to avoid measuring in the dark because pupils may become too large for successful eye tracking (Holmqvist et al., 2011), and because participants may experience strong afterimages if bright visual stimuli are used. Conversely, some other eye trackers (e.g., Crane & Steele, 1985) require large pupils and may thus work better in light-controlled dark rooms. Large changes in pupil size due to changing lighting conditions may also lead to large offsets in the recorded eye tracking data (see, e.g., Wyatt, 2010; Drewes et al., 2012; Choe et al., 2016; Hooge et al., 2021; Hooge et al., in press). One may also wish to avoid incandescent light and other sources of infrared light as these may interfere with the operation of some eye trackers. In our experience, fluorescent and LED lighting are unlikely to interfere with the eye tracker.

Depending on the need for controlled room lighting, it may not be advisable to use a room with windows as an eye tracking lab. Light from outdoors has enormous variation both in terms of intensity and wavelength spectrum throughout the day and throughout the year. Curtains, blinds, and other methods for covering windows may not provide sufficient control over the illumination of the room. Instead, we think that a well thought out setup for an eye tracking lab uses indirect light, ideally provided by some movable lamps that are aimed at the walls or ceilings. If conversely, recordings are made in outdoor conditions, special measures such as visors blocking infrared light (e.g., Matthis et al., 2018) may be used to reduce data loss and improve recording quality. This and other considerations regarding working with uncontrolled lighting conditions during wearable eye tracking recordings are discussed in Fu et al. (2024).

Lastly, depending on one’s needs, one may wish to consider the color of walls and tables in the lab. For instance, light walls and tables may be too reflective if one wishes to work in the dark. For experiments where a completely dark environment is required, special light-proofed rooms can be used (Heywood, 1972; Bennett & Barnes, 2004), in which all surfaces may additionally be painted in flat optical black to prevent any reflections (Shaffer et al., 2003).

## The human factor: Skills and interests

This article so far has considered the tool per se, and has not taken into account the researcher’s context such as team competencies and personal interests. Here we examine this human factor in tool choice, and provide advice for researchers looking to expand their horizons by investing in, for instance, computer or programming skills.

### Choosing tools that fit

An important question a researcher may ask before deciding to adopt a tool in their workflow is whether the tool is suitable *for them*. That is, is the tool needed to address the research question at hand, does it fit within the project’s budget and does the tool fit in one’s workflow and match one’s skills? Let us consider an example eye tracking problem that may be solved with a tool. A researcher, Bobby, is examining gaze behavior while playing a table-top game using a head-worn eye tracker. When drawing up an analysis plan, Bobby runs into the problem that a wearable eye tracker delivers eye movement data referenced to the participant’s head, while for their analysis they need gaze data referenced to the game’s board (cf. Nyström et al., in press). After searching the Internet, Bobby finds a tool that can perform this transformation from head-referenced to world-referenced gaze data for them and an article describing the mathematics implemented by the tool along with a rigorous validation. This looks like just the tool Bobby needs. But can they use it in their project?

To answer this question, it is important to have a clear view of what the competencies present in the research team are, which tools and techniques the members of the team understand, and their motivation. Such an understanding of the team’s skills, limitations and aspirations delineates the solution space available to the team and shows what routes are available for enlarging the solution space if needed. It also allows the team to make strategic decisions about what tools to adopt in the research project. When deciding to use a method or tool in a study, one has a responsibility to have a sufficient understanding of the tool to be able to apply it correctly and explain what one did when reporting the study. In the case of Bobby, who runs their research project by themselves, sadly the tool did not come with a user-friendly GUI. Even though example scripts were provided, Bobby does not know how to read or change such scripts nor has an interest in learning to do so, and as such was not able to adopt the tool into their project.

More concretely, the answer to the question of whether a research team can use a specific tool depends on both the tool and the skills present in the researcher’s team. Some tools are packaged in a convenient graphical user interface and require only minimal computer skills (but may still require sufficient mathematics and physics competence to understand the methods implemented by the tool). Other tools may instead come in the form of a loose collection of files with programming code that the researcher has to adapt for themselves. There are also tools that fall somewhere in between these two extremes, such as tools with well-thought-out programming interfaces and good documentation and examples, but which nonetheless still require some computer or programming skills to be able to adopt them in one’s project. If one finds that the required computer skills, programming skills, or domain knowledge needed to understand and use the tool and method are lacking in the research team, one is left with some options to widen the solution space of the team. These include, for instance,

- Enlisting the help of support staff with the needed technical skills.
- Identifying a willing member of the research team who has the time and interest to invest in the required technical skills.
- Adding an extra co-author to the research team who can bring in the needed skills.

Overall, we think it is important to be realistic when deciding which tools to adopt in an experiment setup and data processing and analysis pipeline. It is easy to underestimate the effort involved in getting a tool or technique to work, especially when the required skills are not available yet. It is also important to be realistic about one’s level of interest in technology. We can imagine that researchers with an interesting research topic may prioritize that research topic over spending a lot of time on technical details such as parsing eye tracker signals or the above-mentioned reference frame transformation for wearable eye tracking data. Instead, the researcher may prefer that some software package takes care of these technical problems without them having to think about it. When roadblocks in terms of insufficient skills or interest are encountered, it may be worth rethinking the study design so that tools that are better suited for the team can be used instead.

### Expanding the solution space: Computer skills and programming

We aim for this article to provide readers with the means to improve the efficiency and quality of the research they perform with eye trackers. This article has hopefully provided the reader with an entry ticket to the vast world of eye tracking tools, but that is only part of the story. Technical skills are another important part of performing effective, high-quality research.

The authors of this article would like to encourage the reader to try to always reserve a little bit of time in the week for improving the technical and other skills (e.g., math, physics, statistics, experimental design, computer skills and programming) that are needed or useful when doing eye-tracking research. In this section we go into examples of how to get started.

Perhaps the ultimate tool of all for a researcher is the computer. For a suitable task and when properly instructed, it can do one’s work, and even the work of an army of research assistants, sometimes in just minutes. This may mean that problems that are impossible to solve manually become possible with a computer. As such, an investment that may improve the effectiveness of one’s research is to invest in computer skills.

For instance, skills using graphical design programs such as Adobe Photoshop and Illustrator, but also The GIMP and Microsoft PowerPoint may be needed to produce the visual stimuli for a study. Skills with Microsoft Excel, SPSS or JASP (JASP Team, 2024) will likely be helpful when organizing and statistically analyzing the eye movement measures produced by tools. For more complicated research projects, further computer skills may be required such as video editing, and perhaps programming skills to enable different tools to work together and to automate mindnumbing repetitive manual tasks. Some studies require specific skills such as programming in languages like Python and MATLAB using toolboxes like PsychoPy (Peirce, 2007; Peirce et al., 2019) and PsychToolbox (Brainard, 1997; Pelli, 1997; Kleiner et al., 2007) to implement custom (and possibly interactive) stimulus presentation routines, or computer vision skills to support the analysis of wearable eye tracking data. Maybe most importantly of all, investing in computer skills allows expanding the solution space of possible study designs, thereby opening new research vistas that are simply not available to researchers who do not have the required skills.

When doing jobs such as building experiments with tens or hundreds of trials, or analyzing data from an eye-tracker that records a thousand samples per second, it is often not an effective use of time to approach this work manually. For these and many other jobs, a good piece of code can vastly improve the effectiveness and efficiency of one’s research compared to performing them manually and also reduces the potential for mistakes to sneak in. Our strong recommendation therefore is for the reader to consider learning to program. What do we mean when we say “learn how to program”? Do we want everybody to become software developers? Of course not. Even only some basic programming skills can already be very useful, allowing a researcher to become more self-reliant and produce more reliable and reproducible results in less time. The authors have time and again seen participants of their introduction to programming courses make such progress.

The level of programming skills that is perhaps easiest to obtain is the ability to take code from the Internet and to be able to set up an environment to get it to run. Also achievable is the ability to read code written by others and understand enough to be able to explain what the code does when writing an article. Perhaps one is also able to make small modifications to adapt the code to one’s own research project, or write small code snippets and insert them in an experiment built with the GUI of tools such as E-Prime or PsychoPy to, for instance, randomize the position of a fixation target. Each of these very obtainable steps in the direction of becoming a proficient programmer may already open up a whole world of new tools and research designs, thereby making new research problems tractable. After all, as shown in this article, many tools in one or another way involve programming. For instance, transforming or converting recorded data to the input format required by the tool almost always requires a tailor-made solution. Often one or two example functions for reading in data are provided with the tool, and only some small changes to one of these functions is enough to enable the tool to read one’s data files, and thereby include the tool in one’s solution space.

While a whole world of new tools already becomes accessible with minimal programming skills, one should consider whether it is worth it to develop programming skills beyond the ability to read and run code written by others and make small modifications. Once one finds out that reading and producing computer code is not as hard as one thought and perhaps even starts to develop a taste for programming, one may start seeing many more possibilities enabled by programming. For instance, it may become enticing to design and write more complicated scripts that can transform one’s data into an analyzable format, and to build a pipeline that processes the data in order with several tools written by others. Lets take an example manual data analysis workflow. It consists of a whole bunch of steps that need to be performed for each recording separately: 1) manually opening each data file in Excel, making a bunch of edits and transformations of columns; 2) importing it in some analysis tool and running that tool; 3) again opening and editing its output in Excel; etc; until finally a file is produced with the relevant eye tracking measures for all participants. A script may reduce this hell of manual data analysis to simply putting the files of recorded eye tracking data in a folder and starting the script. All data analysis steps are automated by the script, and eventually it delivers an output file that is ready for statistical analysis. This level of programming brings many advantages over a manual data analysis workflow, such as:

1. Writing a script forces one to determine *exactly* what data processing steps should be performed. It forces one to design an explicit plan and the script itself provides an exact record of what was done, making it easier to report in scientific publications.
2. Once the script is written, it is simple to make a change at any step in the data processing pipeline. Doing so simply means one has to rerun the script instead of manually performing all the analysis steps again.
3. A script provides a verifiable procedure that enables testing each individual step, and enables examining the impact on a study’s conclusions of changing a parameter or other choice made at any of these steps.

Finally, we get to the terrain of developing one’s own tools, the terrain of software engineers. Developing one’s own tools may well be a relatively niche occupation for scientists using eye tracking. While new tools are sometimes required to be able to address new questions or to make novel discoveries, the majority of scientific discovery unlikely requires new tools, but just an ability to use existing tools in novel ways. Developing one’s own tools, however, does not mean that one is no longer dependent on the tools and platforms of others. For instance, when converting gaze data from pixels on a screen to degrees, one might use existing tools instead of developing one’s own. Building upon the solutions of others and not reinventing everything oneself makes it possible to finish one’s tool in a reasonable amount of time. It, however, also introduces a maintenance burden because new releases of one of the building blocks may introduce a change in behavior (or a bug). One’s own tool would then suddenly no longer work and new investments are needed to fix the situation and restore functionality.

In short, we recommend that researchers who run their own experiments or analyze data consider learning a programming language like Python, R, or MATLAB. For instance, https://software-carpentry.org/lessons/ and https://datacarpentry.org/lessons/ provide relevant courses for learning these languages as well as adjacent best-practice tools and skills (e.g., version control with Git). If you are affiliated with a university, it is possible that you will also be able to locally find an introduction to programming course aimed at non-computer scientists. Many beginners do not invest in learning to program because they, or their supervisors, think they do not have the time for it. We recommend disregarding this short-term argument and finding the time to invest in these skills because it can save you a lot of time later in your research or project. If you heed our advise, the programming language that we would strongly recommend to learn is Python. While there are many great choices such as MATLAB and its PsychToolbox, in our opinion, Python may provide a more flexible and widely applicable choice of programming language for researchers and engineers. Python is one of the most popular programming languages. It is freely available, relatively simple compared to languages like C or Java, and can be a valuable addition to a CV for careers outside academia.

## Conclusion

The goal of this article was to improve the efficiency and quality of research performed with eye trackers by advancing knowledge about all kinds of useful tools. To meet this goal, we have firstly provided an extensive overview of tools that address a wide range of problems in research projects. The aim of this non-exhaustive list was to provide the reader with a sense of the breath of problems for which others have already developed solutions, and as a starting point to finding tools that are suitable for them.

Secondly, we have provided a discussion of how to approach choosing the tools for a research project. A first important factor in choosing the right tool is recognizing good tools. We have discussed what we view as the characteristics of good tools. Once a set of potentially useful (and usable!) tools has been established, one then has to choose which tools to adopt in the research project. This is something that one has to do themselves for each research project because the right choice depends on many factors that we as authors of this article do not know. For instance, what tools are appropriate for a research project depends on the research question, the budget, the available equipment, the skills of the members of the project team, how much time is available and also whether members of the research team even have an interest in spending time on custom tools at all. We have provided a discussion on how to navigate this process and make suitable trade-offs.

A goal of this article was to increase the solution space for researchers, regardless of their level of technical interest and skill, by pointing them to how others have solved problems and helping them gain access to useful tools. We do hope that we have been able to awaken in the reader a little interest in the technical aspects of the work of a researcher. We think there are at least two benefits to having such interests. First, since many researchers are limited in what research question they can explore by the software and other tools they can use, having an interest in exploring new tools or even developing one’s own would broaden one’s research horizons. Second, we think that a research tradition that adopts a single tool as the gold standard may be fragile, as they are at the mercy of mistakes, “smart” tricks and any potential bias in the tool that they use. We think it is useful to look around and explore other tools to see if the main conclusions of one’s work survive when data is collected and/or processed using a different tool.

## Data Availability

Not applicable.

## Footnotes

1 While SMI Experiment Center has not been available since 2017, we suspect it is still widely used.

2 We also keep this list in mind when deciding to publish a tool, since doing so entails going the extra mile and for instance spending time and effort to document the code well, provide good examples, and a detailed readme.

3 It should be noted that “calibration” is technically a misnomer, instead “adjustment” would be the appropriate term for this action (BIPM et al., 2012, p. 28). However, since the term calibration has been in common use among researchers using eye tracking, we will continue using this term throughout this section.

## Declarations

### Open Practices Statement

There are no data or code associated with the present article.
